# Wildlife nidoviruses: biology, epidemiology, and disease associations of selected nidoviruses of mammals and reptiles

**DOI:** 10.1128/mbio.00715-23

**Published:** 2023-07-13

**Authors:** Andrew S. Flies, Emily J. Flies, Nicholas M. Fountain-Jones, Ruth E. Musgrove, Rodrigo K. Hamede, Annie Philips, Matthew R. F. Perrott, Magdalena Dunowska

**Affiliations:** 1 Menzies Institute for Medical Research, University of Tasmania, Hobart, Tasmania, Australia; 2 School of Natural Sciences, University of Tasmania, Hobart, Tasmania, Australia; 3 Healthy Landscapes Research Group, University of Tasmania, Hobart, Tasmania, Australia; 4 Natural Resources and Environment Tasmania, Hobart, Tasmania, Australia; 5 School of Veterinary Science, Massey University, Palmerston North, New Zealand; Albert Einstein College of Medicine, Bronx, New York, USA; Colorado State University, Fort Collins, Colorado, USA

**Keywords:** ssRNA virus, arterivirus, reptile, mammal, emerging infectious disease, serpentovirus, programmed ribosomal frameshift, ICTV, marsupial, wildlife trade, pathology, rehabilitation

## Abstract

Wildlife is the source of many emerging infectious diseases. Several viruses from the order *Nidovirales* have recently emerged in wildlife, sometimes with severe consequences for endangered species. The order *Nidovirales* is currently classified into eight suborders, three of which contain viruses of vertebrates. Vertebrate coronaviruses (suborder *Cornidovirineae*) have been extensively studied, yet the other major suborders have received less attention. The aim of this minireview was to summarize the key findings from the published literature on nidoviruses of vertebrate wildlife from two suborders: *Arnidovirineae* and *Tornidovirineae*. These viruses were identified either during investigations of disease outbreaks or through molecular surveys of wildlife viromes, and include pathogens of reptiles and mammals. The available data on key biological features, disease associations, and pathology are presented, in addition to data on the frequency of infections among various host populations, and putative routes of transmission. While nidoviruses discussed here appear to have a restricted *in vivo* host range, little is known about their natural life cycle. Observational field-based studies outside of the mortality events are needed to facilitate an understanding of the virus-host-environment interactions that lead to the outbreaks. Laboratory-based studies are needed to understand the pathogenesis of diseases caused by novel nidoviruses and their evolutionary histories. Barriers preventing research progress include limited funding and the unavailability of virus- and host-specific reagents. To reduce mortalities in wildlife and further population declines, proactive development of expertise, technologies, and networks should be developed. These steps would enable effective management of future outbreaks and support wildlife conservation.

## INTRODUCTION

Nidoviruses are positive-sense RNA viruses that infect a broad range of animals including terrestrial and marine mammals, fish, birds, reptiles, insects, crustaceans, mollusks, and helminths (1). They share a similar genome organization and expression strategy but vary in the size of genomic RNA and structural components of the virions (2). Nidoviruses are currently classified within the order *Nidovirales*, which includes eight suborders (3). Four suborders (*Abnidovirineae, Mesnidovirineae, Monidovirineae*, and *Ronidovirineae*) contain viruses of invertebrates and one (*Nanidovirineae*) contains viruses of fish. The remaining three suborders (*Arnidovirineae, Cornidovirineae, and Tornidovirineae*) contain vertebrate viruses from fish, reptiles, and mammals. Out of the large group of nidoviruses, only coronaviruses have been so far associated with disease in humans. Human coronavirus infections were associated with mild respiratory disease (4) until 2003, when severe acute respiratory syndrome coronavirus 1 (SARS-CoV-1) emerged in China, followed by the emergence of the Middle East respiratory coronavirus in 2012, and finally SARS-CoV-2 in 2019 (5).

The rate of emerging infectious diseases has risen in recent decades, with most emerging pathogens considered to have zoonotic origins (6 - 8). A transdisciplinary One Health approach that considers the dynamic relationships between people, animals, and their shared environment is needed for the prevention and mitigation of infectious disease outbreaks. Many human-driven activities facilitate contact between humans and wildlife, which in turn may enable spillovers of pathogens to occur either directly from wildlife or indirectly via domesticated animals (9, 10). In addition to the direct impact on human health through cross-species transfer, animal pathogens can exert indirect effects on human populations through the disturbance of existing ecosystems. Broad-scale land use changes and expansion of invasive species are considered the major drivers of biodiversity loss (11). The impact of infectious disease on wildlife is difficult to quantify but is increasingly associated with large-scale species declines (12, 13).

**Fig 1:**
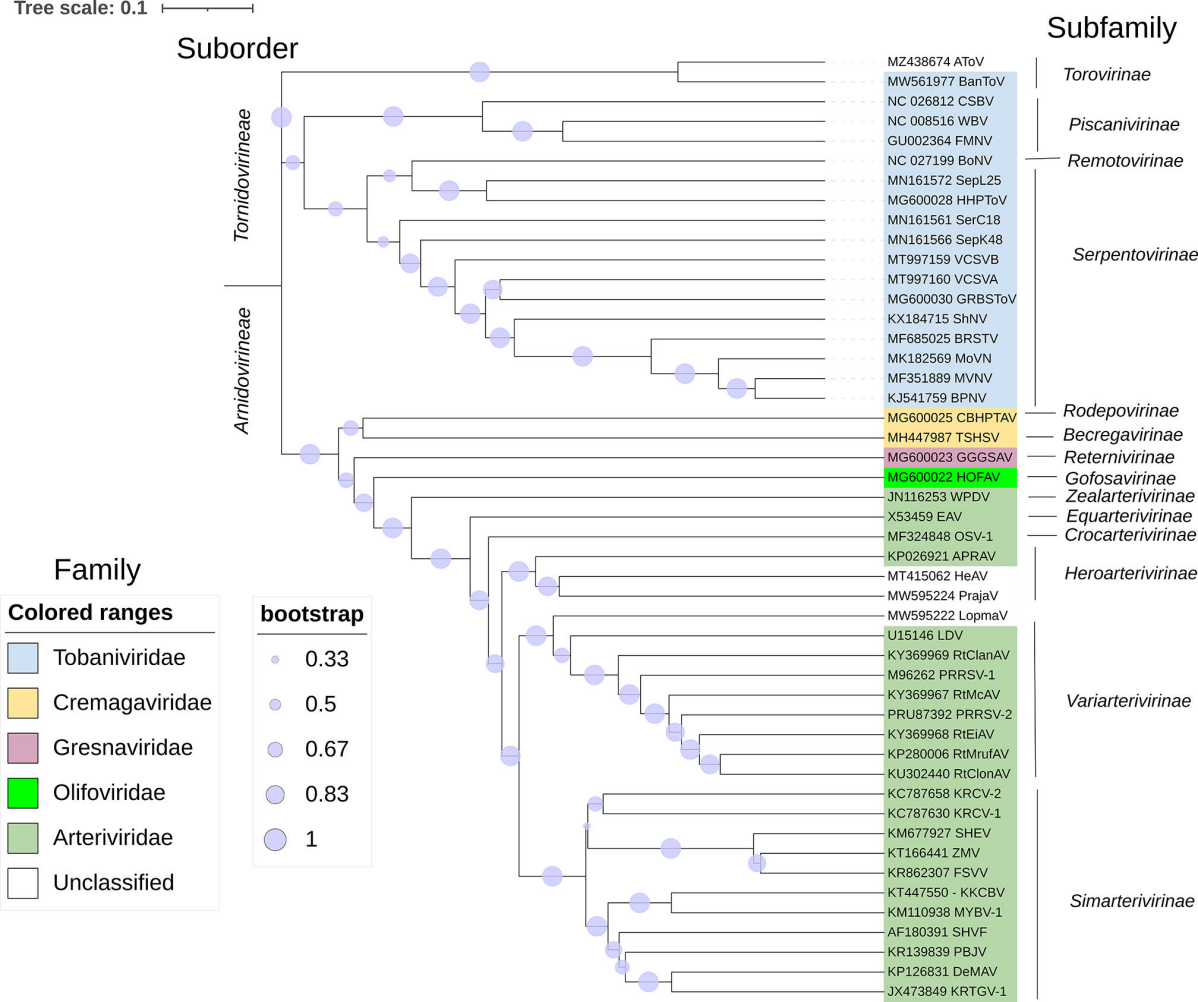
Taxonomy and evolutionary relationships of the selected vertebrate nidoviruses of wildlife from suborders *Tornidovirineae* and *Arnidovirineae*. The evolutionary history was inferred using theunweighted pair group method with arithmetic mean (UPGMA) method based on sequences of open reading frame (ORF) 1b. The accession numbers of sequences used are listed next to each branch. The full virus names and their vertebrate hosts are listed in Table S1. The optimal tree is shown. The proportion of replicate trees in which the associated taxa clustered together in the bootstrap test was calculated from 1,000 replicates. The evolutionary distances were computed using the maximum composite likelihood method and are in the units of the number of base substitutions per site. All ambiguous positions were removed for each sequence pair (pairwise deletion option). There were a total of 9,017 positions in the final data set. Evolutionary analyses were conducted in MEGA11 (18). The tree was visualized using Interactive Tree of Life (iToL) version 6.74 (19).

Several nidoviruses have emerged in wildlife populations within the past decade, sometimes with severe consequences for endangered species. The aim of this minireview was to summarize the key findings from the published literature on nidoviruses of wild mammals and reptiles from two suborders: *Arnidovirineae* and *Tornidovirineae* (Fig. 1). Literature on viruses from the suborder *Cornidovirineae* has not been included, but several reports indicate that there is an abundance of diverse coronaviruses circulating in wildlife (14, 15) and that the natural reservoir hosts for those viruses are warm-blooded flying vertebrates: bats and birds (16). A review on coronaviruses of various hosts, including wildlife, has been recently published (17).

## TAXONOMY

The order *Nidovirales* initially consisted of two families (*Coronaviridae* and *Arteriviridae*), with only four recognized arteriviruses: equine arteritis virus (EAV), porcine reproductive and respiratory syndrome virus (PRRSV), simian hemorrhagic fever virus (SHFV), and lactate dehydrogenase-elevating virus (LDV). New viruses have been discovered and the family *Arteriviridae* is now the largest family in the suborder *Arnidovirineae* (23 species) (Fig. 1). The suborder *Tornidovirineae* contains only one family *Tobaniviridae*, which is further split into four subfamilies, with the largest (*Serpentovirinae*) containing several nidoviruses of reptiles. The subfamily *Torovirinae* contains a recently described torovirus of Tibetan antelope. The remaining two subfamilies of *Tobaniviridae* contain viruses of fish (*Piscanivirinae*) and cattle (*Remotovirinae*), which are outside the scope of this review.

## GENERAL CHARACTERISTICS OF NIDOVIRUSES

Nidoviruses are enveloped viruses with positive-sense single-stranded RNA genomes that are capped at the 5´ end and polyadenylated at the 3´ end (2, 20). The size of nidoviral genomes ranges from 13 kilobases (kb) for arteriviruses (21) to 41 kb for the recently discovered planarian secretory cell nidovirus (PSCN) (22). All nidovirus genomes contain two large overlapping open reading frames (ORF1a and ORF1b) at the 5´ end and several smaller ORFs at the 3´ end. The only exception is PSCN, which was predicted to encode a single large polyprotein from a single ORF (22). The ORF1a and ORF1b are expressed into two large replicase polyproteins through a programmed ribosomal frameshift. These polyproteins are co- and post-translationally auto-cleaved into at least 13 enzymes that are involved in virus replication (23). Replicase subunits that are conserved across all nidoviruses include (from N- to C-terminus) a 3-chymotrypsin-like protease, RNA-dependent RNA polymerase (RdRp), and a Zn-binding domain (ZBD) linked to a helicase (2, 20). The ZBD domain is unique to nidoviruses (2). An uridylate-specific endoribonuclease (NendoU) was also considered to be one of the nidovirus-specific domains until the discovery of insect nidoviruses (family *Mesoniviridae*) which lack this domain (24). Recently, a novel domain named nidovirus RdRp-associated nucleotidyl transferase (NiRAN) has been identified as essential for nidovirus replication (25). Like ZBD, NiRAN has no sequence similarity with proteins outside of the order *Nidovirales* suggesting that these two domains qualify as genetic markers for the order.

The structural and accessory genes are typically expressed through a nested set of 3′ co-terminal subgenomic mRNAs with a common 5′ leader (2, 20). This feature was originally considered to be another defining characteristic of nidoviruses, which gave the order its name (from Latin “nidus,” which means “nest”). It is now apparent that some nidoviruses produce subgenomic RNAs without the leader sequence (26). While there is functional similarity between structural proteins of various nidoviruses, the sequences of those proteins are diverse (21).

## DISCOVERY OF NOVEL NIDOVIRUSES OF WILDLIFE

The accelerating discovery of novel nidoviruses in wildlife has opened the door to better understanding of nidovirus evolution and host range. SHFV was first detected in the 1960s (27 - 29). The existence of several distinct SHFVs was first recognized in 2015 (30). The growing numbers of SHFVs are currently classified into 11 species in the subfamily *Simarterivirinae* (Fig. 1) (21). LDV was first described in 1960 in laboratory mice (31) and later confirmed to exist in wild house mice (32). The first non-LDV arterivirus in rodents was detected in an African pouched rat in Cameroon in 2014 (33). Since then, several other arterivirus sequences have been detected from rodents in different geographical locations (34 - 36).

Rapid expansion of molecular tools allowed for discovery of novel nidoviruses in several other wildlife hosts including shrews (37), hedgehogs (38), marsupials (39, 40), camels (41), and most recently, a protected Tibetan antelope (42). These viruses were identified either during investigations of disease outbreaks (43 - 45) or through molecular surveys of viral diversity in wildlife (34, 35, 46). Reptile nidoviruses were first discovered in 2014 as part of investigations into the etiology of respiratory disease affecting Indian pythons (47) and ball pythons (45, 48). Since then, nidoviruses have been detected in other reptiles including a variety of snakes (49 - 54), lizards (44), chameleons (55), and turtles (43, 56, 57).

It is currently unclear if any of those discoveries represent a recent cross-species transfer as opposed to detection of viruses that had been circulating undiscovered within their natural hosts. The large evolutionary distance between wobbly possum disease virus (WPDV) in marsupials and arteriviruses from placental mammals suggests that WPDV may have separated from other arteriviruses at the time of separation of marsupials into a distinct lineage (58). If so, WPDV was likely introduced to New Zealand with possums from Australia around 1830 and was circulating undetected until 1995, when an outbreak of a fatal neurological disease occurred in a captive research colony (59). It took another 17 years before the etiologically involved nidovirus was reported (39). Similarly, respiratory disease of unknown etiology had been recognized in pythons and Shingleback lizards for years before the discovery of putative etiological nidoviruses (44, 48).

## ASSOCIATION WITH DISEASE

Few of the novel wildlife nidoviruses have been isolated in cell culture and characterized beyond the information derived from the sequence data. This constitutes an important limitation, as it precludes experimental infections with purified material to assess their disease-causing potential.

### Mammals

The original discovery of SHFV was linked to severe hemorrhagic disease among captive macaques, with a total of nearly 300 deaths in two quarantine facilities (27 - 29). Sporadic outbreaks of SHFV among captive macaques continued to occur in various countries (30). Affected animals presented with highly fatal disease characterized by disseminated vascular damage, disseminated intravascular coagulation, and severe hemorrhage in various internal organs (30, 60). It is now recognized that different simian arteriviruses vary in their ability to cause disease in various species of monkeys (61 - 63). In contrast to captive macaques, African monkeys can be infected with SHFV without any overt disease, despite development of long-lasting (years) persistent viremia, and they are hence considered natural hosts for SHFV and similar viruses (62, 64).

In addition to vascular disturbances, neurological deficits have been observed in some SHFV-affected animals (30, 60). Similarly, while infection with LDV is typically subclinical, the virus is neuroinvasive in selected strains of laboratory mice (65). Nidovirus infections of possums and hedgehogs lead to severe neurological disease, although the etiological involvement has been well documented only for wobbly possum disease (WPD) (66). The early clinical signs in possums experimentally infected with WPDV developed after an incubation period of about 2 weeks and included behavioral changes such as subtle intention tremors, increased aggression or sociability, feeding during the day, or loss of appetite (66, 67). This was followed by increased incoordination, and sometimes immobility. The diseased possums lost weight, were unable to climb, and lacked normal defensive behaviors (66, 67). Similar clinical signs were observed in naturally infected possums in New Zealand (68), but the most common presentation of WPD in Australia appeared to be blindness, based on clinical and histopathological data from archival material (40). Only half (3/6) of contemporary possums that met criteria for WPD used in that study tested positive for WPDV RNA (40). Hence, it cannot be excluded that some other pathogens, in addition to WPDV, contribute to the “blindness syndrome” historically reported in Australia.

The potential association of hedgehog arterivirus-1 (HhAV-1) with a fatal neurological disease was proposed based on detection of high levels of viral RNA in samples from wild European hedgehogs that died during a 2019–2020 outbreak of mass mortality in England that involved more than 200 animals (38). However, viral RNA was detected both in tissues with histopathological lesions (brain, liver, spleen) as well as in tissues without apparent histopathological lesions (lungs and livers). In addition, only three hedgehogs were tested for HhAV-1, and hence more data are needed to establish the health implications of HhAV-1 infection in hedgehogs.

### Reptiles

Nidovirus infections of reptiles seem to be mostly associated with respiratory disease of varying severity, although the etiological involvement has been experimentally confirmed only for ball python nidovirus (BPNV) (58). Experimentally infected pythons showed reddening of the choanal and oral mucosa and excessive oral mucus secretion after an incubation period of 4 weeks. The severity of clinical signs increased with time, with excessive swallowing and ventral oral swelling. By 10–12 weeks post-infection, infected snakes developed petechiation of the oral mucosa, increased respiratory distress, open-mouthed breathing, and anorexia. Similar signs were observed in captive pythons naturally infected with serpentoviruses, in addition to other non-specific clinical signs such as shedding skin at unexpected times or spectaculitis (52, 53, 69 - 71). It is possible that factors other than BPNV infection contributed to the expression of disease in some collections, as a small number of BPNV-negative snakes were also clinically affected and bacterial co-infections were common in this (52) and other (45, 48, 50, 53, 54, 70) studies. However, serpentovirus infection was strongly associated with mortalities in snakes that were followed over time (52), and the proportion of serpentovirus-positive animals was significantly higher among diseased compared with clinically healthy captive pythons (54). In contrast, there was no correlation between presence of increased amounts of oral mucous or reddening of oral mucosa and serpentovirus infection among free-ranging Burmese pythons (49), suggesting that factors other than serpentovirus infection may have contributed to clinical signs observed.

The association between nidovirus infection and respiratory disease in other reptiles was suggested based on detection of nidovirus RNA from diseased animals, but experimental infection studies to confirm this are lacking. In one study, 41% of wild Shingleback lizards brought to a wildlife rehabilitation center in Australia with serous or mucopurulent oculonasal discharges were positive for Shingleback nidovirus, compared to 12% of healthy lizards (44). In other studies, novel nidoviruses were detected from dead or diseased animals during outbreaks of mass mortalities involving captive veiled chameleons (55), Bellinger River snapping turtles (43), or farmed *Trionyx sinensis* turtles (57). However, the data available from these outbreaks do not allow these nidoviruses to be implicated as single causes of the mortalities observed. For example, although 23/30 (77%) captive chameleons died of severe respiratory disease over a 13-month period, only two of the clinically affected animals were tested for the presence of veiled chameleon serpentovirus (VCSTV) (55). Both were positive, but so were seven other chameleons, six of which were clinically healthy at the time of euthanasia. The remaining 21 juveniles that died during the outbreak were not tested, presumably because the virus was not yet identified at the time of their death.

Bellinger River snapping turtle virus (BRSTV) was first detected during a catastrophic mass mortality event that involved over 400 endangered turtles in Australia (43). Clinical signs included poor body condition, swollen eyelids, and presence of tan skin foci on the legs of some turtles. A novel serpentovirus was detected from 21 diseased turtles (43). Viral nucleic acids were co-localized with histopathological lesions by *in situ* hybridization in a selection of tissues. While no samples from healthy turtles were tested at the time of the outbreak, only a comparatively small proportion (9/31) of presumably healthy Bellinger River snapping turtles had low levels of BRSTV RNA in a subsequent molecular survey (43). Altogether, this suggests that BRSTV was etiologically involved in the 2015 mass mortality event. However, it cannot be excluded that other pathogens or environmental factors contributed to the severity of disease (43).

## FREQUENCY OF NIDOVIRUS INFECTIONS IN SELECTED WILDLIFE POPULATIONS

Very little data are available on distribution and prevalence of nidovirus infections in wild reptile and mammal populations. Most of these data relied on opportunistic studies in selected geographical locations and cannot be extrapolated to other populations. In addition, most studies relied on detection of the virus, as opposed to detection of antibodies, which is a more reliable approach to assess the prevalence of infection. To exemplify this, the proportion of common brushtail possums (*Trichosurus vulpecula*) positive for WPDV antibodies in Australia (19% of 100 tested) (72) was similar to that reported in New Zealand (20.9% of 230 tested) (73), even though PCR results using the same oligonucleotides yielded strikingly different results for virus detection (72).

Despite these limitations, it appears that nidovirus infections are common among wildlife. Between 19% and 47% of apparently healthy African monkeys from various species were PCR positive for simian arteriviruses (74). Similarly, arterivirus sequences were obtained from three of four clinically normal Olivier’s shrews captured in Guinea over the course of a week at three different locations within a 4 km radius, suggesting that Olivier’s shrew virus 1 (OSV-1) infection was common among Olivier’s shrews at the study site (37). In another study, low levels of BRSTV RNA were detected in oral or conjunctival swabs from 29% of 31 apparently healthy Bellinger River snapping turtles and 4% of 49 Murray River turtles (43).

Nidovirus infection seems to be common in captive pythons with similar numbers of nidovirus-positive pythons reported in two European studies (27% of 95 and 31% of 1,426 snakes) (51, 54) and in one USA-based study (38% of 414 snakes) (52). A slightly lower (20% of 5,210 snakes) prevalence was reported among pet snakes from various European countries between 2016 and 2021 based on data from a commercial veterinary laboratory, which likely reflects selection bias (75). Green tree pythons (51, 54) and Indian pythons (75) were the most frequently infected among species represented by more than 10 individuals. Considerably lower infection rates were reported for boas (2.4%–10%) and colubrids (0.9%), with no positive snakes from a relatively small number of other families tested (51, 52). The frequency of serpentovirus infection among free-ranging pythons was investigated only in one study of invasive Burmese pythons in Florida and was only slightly lower (24% of 172 snakes) than the numbers reported from captive collections (49).

## GROSS AND HISTOPATHOLOGICAL FINDINGS, TISSUE TROPISM

Gross and histopathological changes reported varied between viruses and between wildlife hosts. Proliferative interstitial pneumonia, referred to as “nidovirus associated proliferative disease” was the most consistent pathological finding for serpentovirus-infected snakes (52, 71). Some pythons also showed pathology in the upper respiratory tract (rhinitis or tracheitis) and in the gastrointestinal tract (stomatitis, esophagitis) (71). Necropsies of BRSTV-positive turtles revealed general poor body condition, swollen eyelids, and tan foci on the skin of inner thighs (43). Three HhAV-1-positive hedgehogs (38) and two VCSTV-affected chameleons (55) had no gross pathological lesions. Consistent gross pathological findings in WPD-affected possums included loss of fat reserves and a variable degree of muscle wasting (66, 67, 76, 77). Enlargement of the kidney, liver, spleen, and lymph nodes was apparent in a subset of infected possums. No gross lesions were identified in pouch young from experimentally infected mothers.

The neurological signs observed in WPD-affected possums are likely to reflect the lesions of the central nervous system including gliosis, meningoencephalitis, and the presence of glial nodules (39, 76). Non-suppurative inflammation, with a dominance of lymphocytes and plasma cells, was commonly seen in the meninges and brain parenchyma, as well as in the liver, spleen, and kidney, often in association with blood vessels. The presence of viral RNA was demonstrated within these lesions using immunohistochemistry (39, 76). Similar histopathological lesions were described for possums in Tasmania, but possums presumably affected by WPD on mainland Australia typically showed lesions only in the brain and the eyes (40). Perivascular cuffing containing small numbers of neutrophils intermingled with microglia and mononuclear cell infiltrates was observed in brains of HhAV-1-infected hedgehogs. The affected animals had several other lesions in the brains, as well as in the kidneys and spleens (38). Vascular lesions (fibrinoid vasculopathy) were also reported in a proportion of BRSTV-affected turtles (43). Heterophilic colitis was the only common finding in two VCSPV-affected chameleons with one of the animals also showing several histopathological changes in the respiratory system (55). Splenic and nephric inflammation and necrosis in addition to peri-orbital histopathology were histopathological features of hedgehogs positive for HhAV-1 (38).

**Fig 2:**
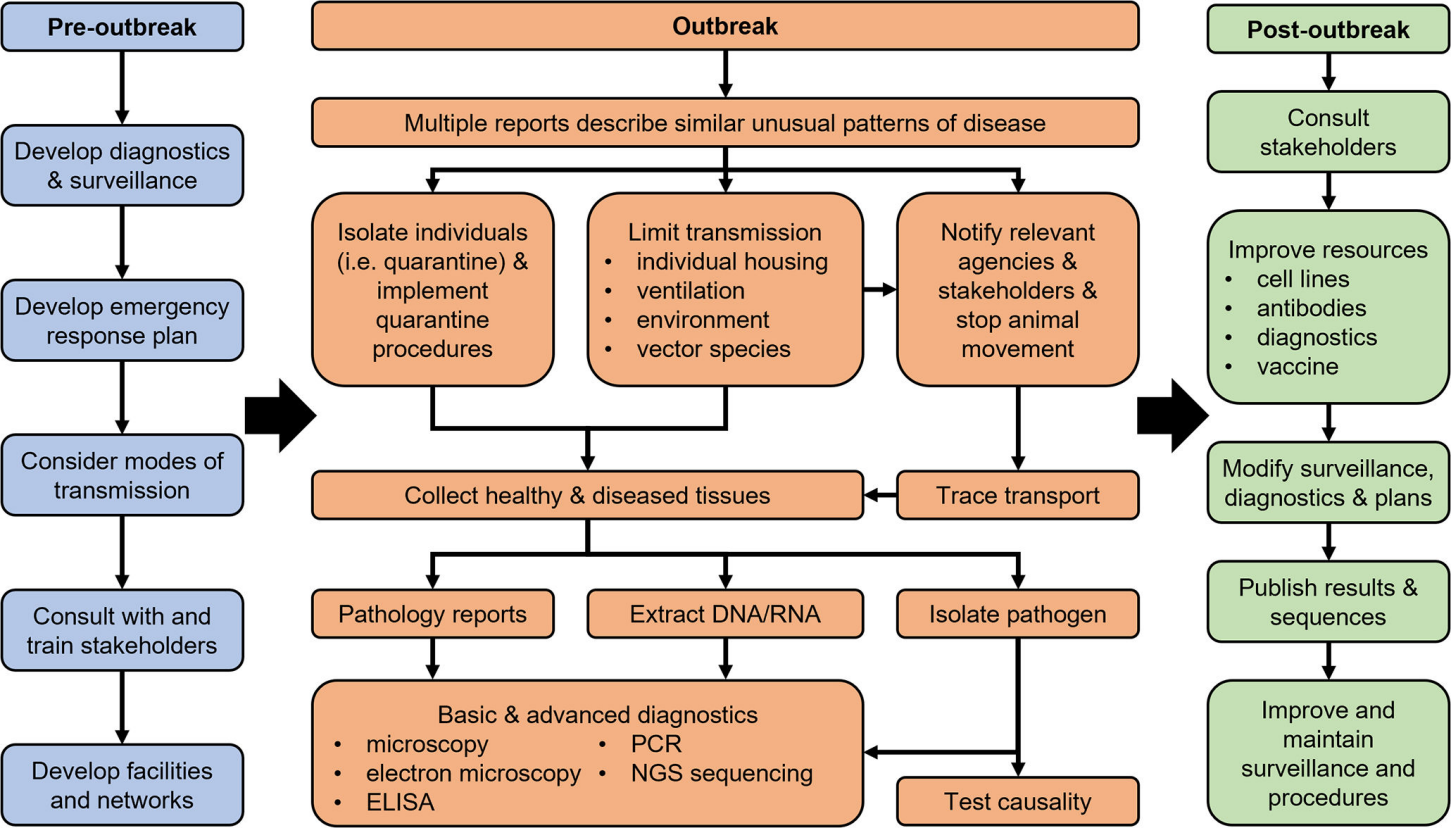
Overview of basic response principles for outbreaks of infectious disease among wildlife. Separate but linked plans need to be in place for pre-outbreak preparedness, outbreak response procedures, and post-outbreak follow-up. Strategies for outbreaks of known pathogens and novel pathogens should be in place for any group involved in wildlife activities. An outbreak response is triggered by multiple reports of unusual signs of disease, with a higher alarm level if reports are from independent sources. Key initial response elements are to isolate infected individuals and limit further spread. The hunt for the potential disease-causing virus or other agent ensues. Infrastructure, reagents, and funding are often limiting factors that can be mitigated through preparedness, planning, and development of research and management networks. Flow chart based on information in WHO and World Organisation for Animal Health reports (78, 79).

Nidoviruses tend to have a broad tissue distribution, even if clinical signs are localized predominantly to one body system. Viral RNA was detected in all tissues examined from BRSTV-affected turtles (43), WPD-affected possums (76), and two of three arterivirus-positive shrews (37). The types of tissues tested varied between studies, but all included lung, spleen, kidney, and liver. Serum, brain, lymph nodes, urine, heart, eye, and ovary were additionally tested in some studies. The broad tissue distribution likely reflects the cellular targets of these viruses. Arteriviruses infect cells of monocytic/macrophage lineage (21), which would explain the presence of these viruses in a variety of tissues, and high levels of viral RNA in tissues rich in lymphatic cells such as liver or spleen. Permissiveness of different cell types *in vivo* for nidovirus replication likely differs between viruses, as snake serpentoviruses have been detected in a broad range of cells including hepatocytes, renal tubules, pancreatic ducts, endothelial cells, and monocytes (71). Investigations of cellular tropism of wildlife nidoviruses are hindered by lack of virus- and host-specific reagents (Fig. 2).

## TRANSMISSION

Considering the fragile nature of most enveloped viruses, it seems likely that close contact (either direct or through shared environment) is required for efficient transmission of wildlife nidoviruses (Fig. 2), at least among terrestrial hosts. Experimental transmission studies performed with WPDV seem to support this view (67). All healthy adult possums in the group enclosure with diseased possums contracted WPD, but healthy possums kept in cages closely adjacent to cages with diseased possums remained healthy. In contrast, serpentoviruses spread from quarantined to resident snakes when both collections shared a common airspace, although the contribution of fomites cannot be excluded (52).

Experimentally, WPDV was transmitted via several routes (intra-peritoneal, intra-dermal, intra-gastric, intra-tracheal, and intra-dermal) with a variety of infectious materials including homogenized tissue suspension, urine, blood, or homogenized mites collected from WPD-affected possums (67). It was hypothesized that under natural conditions, transmission could occur through activities such as fighting and grooming, and possibly also mechanically through insect bites or from environmental contamination in areas with high density of possums. The presence of WPDV in semen and the possibility of sexual transmission have not been investigated. This is an important route of transmission for EAV (80) and should be considered as a possible route of spread of WPDV and other wildlife arteriviruses (Fig. 2).

Experimental infection of pythons with BPNV was achieved through oral or intra-tracheal administration of the virus (58). The highest levels of viral RNA were detected in the respiratory and gastrointestinal tracts, suggesting that under natural conditions, transmission through both close contact (respiratory route) or environmental contamination (fecal-oral route) may occur. Cloacal, conjunctival, and mouth swabs from the diseased Bellinger River snapping turtles were positive for viral RNA suggesting that the turtles shed the virus via multiple routes (43). The aquatic habitat of these turtles provides an environment that may have facilitated dispersal of the virus.

Both PRRSV and EAV can be transmitted vertically and are important causes of abortion in pigs and horses, respectively (81, 82). The joeys of WPDV-infected mothers were often infected with the virus, but it was not established whether it was due to horizontal transmission versus vertical transmission via milk (66, 67). Eggs and hatchlings of BPNV-positive pythons were mostly negative for BPNV suggesting that vertical transmission is not a common feature of serpentovirus infection in pythons (52). It remains to be established whether other wildlife nidoviruses can be transmitted vertically via placenta (mammals) or eggs (reptiles) and with what consequences for the offspring.

### Persistence

Persistent arterivirus infections have long been recognized in EAV-infected stallions (80, 83), LDV-infected mice (84), and PRRSV-infected swine (85), although the sites and mechanisms of persistence differ between viruses. Simian arteriviruses establish persistent subclinical infections with high-level viremia in African monkeys including patas monkeys, vervets, or baboons (62, 64). None of the possums experimentally infected with WPDV cleared the virus before death or euthanasia approximately 3–4 weeks post-infection (66, 76). However, tissues from serologically positive wild possums were commonly negative for the virus indicating that either WPDV does not establish long-term persistence in possums or persistence occurs in tissues that were not sampled (72, 73). Limited data available suggest that some tornidoviruses also establish persistent infections in their hosts. Serpentoviruses were consistently detected in oral swabs from naturally infected captive pythons that were sampled on multiple occasions for up to 28 months (52).

### Are vectors involved in nidovirus transmission?

Several different nidoviruses have been detected in mosquitoes (86, 87). The mosquito-borne nidoviruses cluster together separately from other nidoviruses and are currently classified in the family *Mesoniviridae* (3, 24). It is assumed that they are unlikely to infect vertebrate hosts, based on their inability to grow in any of the vertebrate cell lines tested, although experimental infection studies or serological surveys have not been reported (87).

In a recent study, a sequence of a torovirus-like virus was detected from a pool of ticks collected from camels in Africa and named Bangali torovirus (BanToV) (41). The follow-up serological testing showed that 6/59 (10%) of camels tested contained antibodies against the predicted nucleocapsid protein of BanToV, suggesting than BanToV is transmitted between ticks and camels (41). Similarly, WPDV was successfully transmitted from diseased to healthy possums via transdermal inoculation of homogenized mites collected from WPD-affected possums suggesting that biting insects may play a role in transmission of the virus (67). Elucidating if hematophagous invertebrates such as mosquitoes, midges, and ticks can serve as vectors for nidoviruses would help efforts to manage nidoviral diseases in wildlife (Fig. 2).

### Species specificity and zoonotic potential

Nidoviruses vary in their species specificity. The emergence of SARS, Middle East respiratory syndrome (MERS), and SARS-CoV-2 in people and reverse-zoonotic transmission of SARS-CoV-2 to a variety of animals demonstrate that coronaviruses can cross species barriers (88). In contrast, equine and porcine arteriviruses tend to have a narrow host range that includes only animals from closely related species (21). Arteriviruses of wildlife seem to follow that trend. Simian arteriviruses from various species of African monkeys share only about 50% nucleotide identity with one another, suggesting that different viruses circulate among monkeys of different species (74). Similarly, rodent arteriviruses have likely co-evolved with their hosts, as most viruses analyzed clustered together in lineages that were phylogenetically consistent with the lineages formed by their hosts (34, 35). However, the closest known relatives of Praja virus of mice were HhAV-1 and African pouched rat arterivirus. The close phylogenetic relationships between arteriviruses from distantly related hosts may suggest a recent host-switching event, although a long-term convergent evolution could also produce similar results (35).

The available data suggest that tornidoviruses also have a narrow host range. Only two species of turtles tested positive for BRSTV during a post-outbreak molecular survey, and 422 samples from more than 20 other species were all negative for BRSTV RNA (43). Serpentoviruses were detected only in veiled chameleons, and in none of the other lizards housed in the same facility (55). Although monophyletic clades by host were not well supported in the phylogeny of serpentoviruses detected in several collections of captive snakes, viruses with more similar sequences were generally found in snakes of the same species or genus (52). Nearly identical sequences were, however, occasionally found in pythons from different species. However, none of the captive snakes from *Lamprophiidae*, *Elapidae*, and *Viperidae* from collections with serpentovirus-positive pythons tested positive for serpentovirus RNA. Similarly, only 5/219 (2%) samples collected from free-ranging native snakes in Florida tested positive for serpentovirus RNA, as opposed to 42/172 (24%) of free-ranging pythons in the same region (49). The genomes of serpentoviruses from native snakes were divergent from those detected in pythons, indicating that they were infected with different viruses.

The relative host specificity of arnidoviruses and tornidoviruses suggests that the risks of their zoonotic transmission are comparatively low. To support this view, these viruses have so far never been detected from the human host (89). However, some biological features of these viruses such as high prevalence, high variability, and the ability to establish persistent infections may facilitate cross-species transfer, particularly of simian arteriviruses from non-human primates (74). Additionally, cross-species transfer of simian arteriviruses has already been documented during the original outbreaks of severe hemorrhagic disease in macaques, and SHFV can replicate in human monocyte cultures *in vitro* (90).

The potential zoonotic risks posed by other arnidoviruses or tornidoviruses are most likely low, but should be considered in wildlife management plans and captive animal husbandry practices (Fig. 2), particularly for wildlife hosts that live in proximity to humans and farm animals, such as rodents. The risk of cross-species transmission is also increased in any situation when different wildlife species aggregate. Such conditions are created during the global trade of illegal wildlife, with mammals and reptiles being commonly trafficked, often in poor husbandry conditions (91). Transport of wild animals to farms or urban centers also brings into contact animals from species that do not normally interact with each other. High-density animal farms pose similar risks, exemplified by cross-species transmission of SARS-CoV-2 between humans and mink (*Neovison vison*) at mink farms (92). Wildlife hospitals and rehabilitation centers may also inadvertently facilitate spillover events, as staff are regularly in close contact with animals from multiple species.

## LABORATORY TESTS FOR DETECTION OF NIDOVIRUS INFECTIONS

The common diagnostic approaches to identify novel viruses include the use of electron microscopy, virus isolation in cell culture, and various molecular techniques for detection of viral DNA/RNA (Fig. 2). Most of the viruses described in this review have been originally discovered using next-generation sequencing with nucleic acids extracted directly from tissues of affected animals (39, 44, 45, 53, 93). Occasionally, transmission electron microscopy was used to help identify viruses present in clinical samples (43, 94). Sequence data of BRSTV were obtained after the initial isolation of the virus in culture African green monkey kidney cells (43). Other molecular techniques including random PCR, degenerate PCR with broadly reactive primers, genome walking, or 5´/3´ rapid amplification of cDNA ends (RACE) have also been employed for detection of novel nidoviruses and determination of full genomic sequence, often in combination with next-generation sequencing (NGS) (39). *In situ* hybridization or immunohistochemistry were used to co-localize WPDV (39, 76), snake serpentoviruses (71), and BRSTV (43) with histopathological lesions.

Virus-specific RT-PCR assays have been used to determine the frequency of infection among selected populations. Typically, those assays targeted highly conserved regions of ORF1b (39, 43, 44, 53). However, even primers from conserved genomic regions may fail to detect all related viruses, as can be exemplified by variable sensitivity of different primers for detection of divergent snake serpentoviruses (49, 52). This raises the possibility that the overrepresentation of pythons among serpentovirus-infected snakes in various studies reflects a diagnostic bias linked to the use of primers targeting sequences of python viruses. The detection of diverse nidovirus sequences from snakes in a recent metagenomic study in China seems to support this view (1, 46). Unlike python serpentoviruses (suborder *Tornidovirineae*), these novel nidoviruses clustered in the suborder *Arnidovirineae* and were classified into newly created families: *Gresnaviridae* and *Olifoviridae* (Fig. 1).

The ability to grow a novel virus in cell culture enables further characterization of such virus and facilitates the development of serological assays. Except for EAV, arteriviruses tend to have very restricted *in vitro* host range (21). Isolation in cell culture has been mostly successful only in primary cells of macrophage lineages from the natural hosts (95 - 98). Some of the SHFV and PRRSV isolates were also adapted to grow in a monkey kidney cell line (MA-104) and its subclones (e.g., MARC-145) (61, 99), but primary possum macrophages remain the only supportive cells for culture of WPDV (95). Since primary cell cultures are difficult to establish and maintain, availability of continuous cell lines that support the growth of arteriviruses would be desirable. One strategy to generate such cells is to genetically modify existing cell lines by introduction of CD163, which is expressed on macrophages and monocytes and has been shown to be a primary receptor for some arteriviruses (90, 100, 101). Such approach has been already successfully used for PRRSV (102, 103).

Tornidoviruses appear to grow in a broader selection of cells compared with arnidoviruses. Serpentoviruses have been grown in a variety of primary cells derived from various species of snakes including heart, kidney, liver, brain, and lung (53, 58). BRSTV grew in monkey cell lines (Vero, BGM (RRID:CVCL_4125), and CV-1 (RRID:CVCL_0229)) with cytopathic effect and in bovine kidney cell line (MDBK (RRID:CVCL_0421)) without cytopathic effect (43). However, VCSTV did not grow in the cell lines tested including primary cells from boa constrictor kidney and diamond python heart, as well as continuous cell lines from iguana (IgH2) and viper (VH2) hearts (55).

Development of serological tests for detection of antibodies against novel wildlife viruses is challenging due to lack of species-specific reagents, lack of data on the main viral targets for antibody production in virus-infected wildlife, as well as difficulties with sourcing known-negative sera. Nonetheless, an indirect enzyme-linked immunosorbent assay (ELISA) with recombinant protein N and luciferase immunoprecipitation system were developed to allow serological testing for WPDV and BanToV infections, respectively (41, 73). The development of tests for detection of virus-specific antibodies in reptiles is further complicated by the comparatively limited knowledge of reptiles’ immune responses as compared to mammals (104). Perhaps not surprisingly, serological assays for detection of nidovirus infections in reptiles have not yet been developed (105).

## OUTBREAK RESPONSE AND CONTROL

Treatment of infected wildlife is difficult and often not feasible. Reported regimens consisted of supportive care (broad-spectrum antibiotics, nebulization, rehydration), with variable success (43, 71). The key element to effective control of an outbreak of infectious disease in wildlife is prevention (Fig. 2). Appropriate preparedness (availability of resources, expertise, and plans) can greatly facilitate diagnostic investigations and implementation of control measures during the outbreak. It is also important to disseminate the knowledge gained and apply long-term management changes to minimize the impact of a given pathogen on the health of the animals once the outbreak is controlled.

A quarantine period should be observed whenever possible when translocating wild animals into captivity or moving captive animals between different facilities. Physical separation (separate buildings) combined with other infection control measures (the use of separate clothes, shoes, equipment, one-way flow of bedding and feeding, shower-out, and routine disinfection of hands and surfaces) were effective at controlling the spread of serpentovirus infections among captive pythons (52): 31/33 (94%) of quarantined snakes were serpentovirus-positive, while only 1/25 snakes in the main collection contracted the virus (52). Similarly, the frequencies of infection in another captive collection where rigorous quarantine procedures were implemented were 26/38 (68%) and 5/117 (4%) for the quarantined and main collections, respectively (52). Where full separation of staff and equipment is not feasible, sick animals should be handled last, after attending to healthy and injured animals, with rigorous cleaning and disinfection of clothing, hands, and equipment. To achieve this, basic training in wildlife disease and biosecurity, such as those provided by Wildlife Health Australia (106) and the World Organization for Animal Health (107), should be provided to all staff at any facility where wildlife is handled.

Implementing any type of control measures for the illegal wildlife trade is extremely challenging. However, illegal trade is fueled by profit, which requires animals to be alive and relatively healthy. Thus, online information sheets outlining best practices tailored for trafficked species might help improve animal welfare and reduce risk of transporting diseased animals.

Control efforts summarized in Fig. 2 are easier to apply to captive wildlife than free-living wildlife. While several of the disease outbreaks described here occurred among captive animals (48, 55, 59), some occurred among animals in their natural environment (38, 43). Factors other than those associated with captivity (e.g., high density of susceptible animals, stress, use of shared equipment and air space, and common food sources) must have affected the host-pathogen balance and precipitated those outbreaks. The triggers that lead to mass mortality events in free-living wildlife are likely to be complex and extend beyond the presence/absence of a specific pathogen in the animals’ environment. Understanding the relationships between the pathogen, the host, and the environment is essential for conservation of the existing ecosystems. Such knowledge may also facilitate interventions for effective control of an outbreak, should it occur. Both laboratory-based studies to understand the pathogenesis of diseases caused by novel pathogens as well as observational field-based studies outside of the mortality events to understand the prevalence, transmission, inter-relationships, and impact of endemic infections are necessary to derive the relevant data. Wealth, language, geographical location, and security were identified as key barriers to collection of global biodiversity data, which is a prerequisite for any conservation efforts (108). Any research into viruses of wildlife faces similar challenges, which can be partly resolved by recognition of the One Health principles, increased funding, and effective international collaborations.

## CONCLUSIONS

Rapidly advancing sequencing technologies have facilitated the discovery of novel nidoviruses. However, most of those viruses are poorly characterized beyond the sequence data. Some general assumptions about those viruses are based on characteristics of a few comparatively better-studied exemplars and may prove inaccurate for others. One example may be host specificity. While nidoviruses described above appear to be largely host-specific, the possibility of host switching under favorable conditions cannot be excluded. Most of the data currently available are sporadic and fragmented. Nonetheless, it appears that nidovirus infections are common among wildlife. Proactive development of reagents for the *in vitro* and *in vivo* studies would facilitate further research and characterization of nidoviruses of wildlife. Regular communication between government organizations, researchers, and wildlife rehabilitation centers is needed to ensure early detection and rapid responses to novel diseases. Establishment of archives of material from field-based studies would facilitate tracing viral lineages. This would help to elucidate the evolutionary pathways of novel nidoviruses and putative sources for pathogens involved in the outbreaks. Limited availability of funding for wildlife research is one of the key factors preventing more rapid advancement of knowledge in this area. The increasing recognition of the interconnection between wildlife and human health (One Health concept) will hopefully remedy this in the future.

## SUPPLEMENTAL MATERIAL

Table S1 mbio.00715-23-s0001.pdf Taxonomic classification and hosts of selected nidoviruses of vertebrate hosts in the suborders Arnidovirideae and Tornidovirideae, including exemplar GenBank accession numbers.

ASM does not own the copyrights to Supplemental Material that may be linked to, or accessed through, an article. The authors have granted ASM a non-exclusive, world-wide license to publish the Supplemental Material files. Please contact the corresponding author directly for reuse.
