# Supplementary material for: Wildlife nidoviruses: biology, epidemiology, and disease associations of selected nidoviruses of mammals and reptiles
Source: mBio. 2023 Jul 13;14(4):e00715-23. doi: 10.1128/mbio.00715-23 (PMC10470586; doi:10.1128/mbio.00715-23)
Supplement: Table S1 — Taxonomic classification and hosts of selected nidoviruses of vertebrate hosts in the suborders Arnidovirideae and Tornidovirideae, including exemplar GenBank accession numbers. [file mbio.00715-23-s0001.pdf]

SUPPLEMENTAL MATERIAL

TITLE

Wildlife nidoviruses: Biology, epidemiology, and disease associations of selected nidoviruses in mammals and reptiles

RUNNING TITLE

Nidoviruses in wild mammals and reptiles

AUTHORS

Andrew S. Flies,[a] Emily J. Flies,[b,c] Nicholas M. Fountain-Jones,[b] Ruth E. Musgrove,[a] Rodrigo K. Hamede,[b], Annie Philips,[d] Matthew R.F. Perrott,[e] Magdalena Dunowska[e]

AFFILIATIONS

[a]Menzies Institute for Medical Research, University of Tasmania, Hobart, TAS 7000, Australia
[b]School of Natural Sciences, University of Tasmania, Hobart, TAS 7001, Australia
[c]Healthy Landscapes Research Group, University of Tasmania, Hobart, TAS 7001, Australia
[d]Natural Resources and Environment Tasmania, Hobart, TAS 7000, Australia.
[e]School of Veterinary Science, Massey University, Palmerston North, New Zealand.

CORRESPONDING AUTHOR

Andrew S. Flies, andy.flies@utas.edu.au

KEYWORDS

ssRNA virus; arterivirus; reptile; mammal; emerging infectious disease; serpentovirus; programmed ribosomal frameshift; ICTV; marsupial; wildlife trade; pathology; rehabilitation

Supplemental Table 1: Taxonomic classification and hosts of selected nidoviruses of vertebrate hosts in the suborders *Arnidovirideae* and *Tornidovirideae* including exemplar GenBank accession numbers.

Family	*Subfamily*	*Genus*	*Species*	Virus name	Virus name Abbreviation	Host (family)	Accession
			**Suborder *Arnidovirineae***				
Arteriviridae	*Crocarterivirinae*	*Muarterivirus*	*Muarterivirus afrigant*	Olivier's shrew virus 1	OSV-1	*Soricidae*	MF324848
Arteriviridae	*Equarterivirinae*	*Aphaarterivirus*	*Alphaarterivirus equid*	equine arteritis virus	EAV	*Equidae*	X53459
Arteriviridae	*Heroarterivirinae*	*Lambdaarterivirus*	*Lambdaarterivirus afriporav*	African pouched rat arterivirus	APRAV	*Nesomyidae*	KP026921
Arteriviridae	*Heroarterivirinae*[a]	*unclassified*	*unclassified*	hedgehog arterivirus-1	HhAV-1	*Erinaceidae*	MT415062
Arteriviridae	*Heroarterivirinae*[b]	*unclassified*	*unclassified*	PrajaV	PrajaV	*Muridae*	MW595224
Arteriviridae	*Simarterivirinae*	*Deltaarterivirus*	*Deltaarterivirus hemfev*	simian haemorrhagic fever virus	SHFV	*Cercopithecidae*	AF180391
Arteriviridae	*Simarterivirinae*	*Epsilonarteriviru*	*Epsilonarterivirus hemcep*	simian haemorrhagic encephalitis virus	SHEV	*Cercopithecidae*	KM677927
Arteriviridae	*Simarterivirinae*	*Epsilonarteriviru*	*Epsilonarterivirus safriver*	Free State vervet virus	FSVV	*Cercopithecidae*	KR862307
Arteriviridae	*Simarterivirinae*	*Epsilonarteriviru*	*Epsilonarterivirus zamalb*	Zambian malbrouck virus 1	SMbV-1	*Cercopithecidae*	KT166441
Arteriviridae	*Simarterivirinae*	*Etaarteriviru*	*Etaarterivirus ugarco 1*	Kibale red colobus virus 2	KRCV-2	*Cercopithecidae*	KC787658
Arteriviridae	*Simarterivirinae*	*Iotaarteriviru*	*Iotaarterivirus debrazmo*	DeBrazza's monkey arterivirus	DeMAV	*Cercopithecidae*	KP126831
Arteriviridae	*Simarterivirinae*	*Iotaarteriviru*	*Iotaarterivirus kibreg 1*	Kibale red-tailed guenon virus 1	KRTGV-1	*Cercopithecidae*	JX473849
Arteriviridae	*Simarterivirinae*	*Iotaarteriviru*	*Iotaarterivirus pejah*	Pebjah virus	PBJV	*Cercopithecidae*	KR139839
Arteriviridae	*Simarterivirinae*	*Thetaarterivirus*	*Thetaarterivirus kafuba*	Kafue kinda chacma baboon virus	KKCBV	*Cercopithecidae*	KT447550
Arteriviridae	*Simarterivirinae*	*Thetaarterivirus*	*Thetaarterivirus mikelba 1*	Mikumi yellow baboon virus 1	MYBV-1	*Cercopithecidae*	KM110938
Arteriviridae	*Simarterivirinae*	*Zetaarteriviru*	*Zetaarterivirus ugarco 1*	Kibale red colobus virus 1	KRCV-1	*Cercopithecidae*	KC787630
Arteriviridae	*Variarterivirinae*	*Betaarteriviru*	*Betaarterivirus suid 1*	porcine reproductive and respiratory syndrome virus 1	PRRSV-1	*Suidae*	M96262
Arteriviridae	*Variarterivirinae*	*Betaarteriviru*	*Betaarterivirus suid 2*	porcine reproductive and respiratory syndrome virus 2	PRRSV-2	*Suidae*	U87392
Arteriviridae	*Variarterivirinae*	*Betaarteriviru*	*Betaarterivirus ninrav*	RtClon areterivirus	RtClonAV	*Muridae*	KU302440
Arteriviridae	*Variarterivirinae*	*Betaarteriviru*	*Betaarterivirus sheoin*	RtEi arterivirus	RtEiAV	*Muridae*	KY369968
Arteriviridae	*Variarterivirinae*	*Betaarteriviru*	*Betaarterivirus timiclar*	RtMc arterivirus	RtMcAV	*Muridae*	KY369967
Arteriviridae	*Variarterivirinae*	*Betaarteriviru*	*Betaarterivirus chinrav 1*	RtMruf arterivirus	RtMrufAV	*Muridae*	KP280006
Arteriviridae	*Variarterivirinae*	*Nuarterivirus*	*Nuarterivirus guemel*	RtClan artervirus	RtClanAV	*Muridae*	KY369969
Arteriviridae	*Variarterivirinae*	*Gammaarterivirus*	*Gammaarterivirus lacdeh*	lactate dehydrogenase-elevating virus	LDV	*Muridae*	U15146
Arteriviridae	*Variarterivirinae*[b]		*unclassified*	LopmaV	LopmaV	*Muridae*	MW595222

Arteriviridae	*Zealarterivirinae*	*Kappaarterivirus*	*Kappaarterivirus wobum*	wobbly possum disease virus	WPDV	*Phalangeridae*	JN116253
Cremegaviridae	*Becregavirinae*	*Sicregavirus*	*Sicregavirus nixi*	Trionyx sinensis hemorrhagic syndrome virus	TSHSV	*Trionychidae*	MH447987
Cremegaviridae	*Rodepovirinae*	*Pontunivirus*	*Chinturpovirus 1*	Chinese broad-headed pond turtle arterivirus	CBHPTAV	*Geoemydidae*	MG600025
Gresnaviridae	*Reternivirinae*	*Cyclophivirus*	*Ptyasnivirus 1*	Guangdong greater green snake arterivirus	GGGSAV	*Colubridae*	MG600023
Olifoviridae	*Gofosavirinae*	*Kukrinivirus*	*Oligodon snake nidovirus 1*	Hainan oligodon formosanus arterivirus	HOFAV	*Colubridae*	MG600022
			**Suborder *Tornidovirineae***				
Tobaniviridae	*Piscanivirinae*	*Bafinivirus*	*White bream virus*	white bream virus	WBV	*Cyprinidae*	NC_008516
Tobaniviridae	*Piscanivirinae*	*Bafinivirus*	*Fathead minnow nidovirus 1*	fathead minnow nidovirus	FMNV	*Cyprinidae*	GU002364
Tobaniviridae	*Piscanivirinae*	*Oncotshavirus*	*Chinook salmon nidovirus 1*	Chinook salmon bafinivirus	CSBV	*Salmonidae*	NC_026812
Tobaniviridae	*Remotovirinae*	*Bostovirus*	*Bovine nidovirus 1*	bovine nidovirus	BoNV	*Bovidae*	NC_027199
Tobaniviridae	*Serpentovirinae*	*Lyctovirus*	*Lyctovirus alpa*	veiled chameleon serpentovirus A	VCSTV-A	*Chamaeleonidae*	MT997160
Tobaniviridae	*Serpentovirinae*	*Pregotovirus*	*Ball python nidovirus 1*	Ball python nidovirus	BPNV	*Pythonidae*	KJ541759
Tobaniviridae	*Serpentovirinae*	*Pregotovirus*	*Berisnavirus 1*	Bellinger River snapping turtle virus	BRSTV	*Testudines*	MF685025
Tobaniviridae	*Serpentovirinae*	*Vebetovirus*	*Vebetovirus paba*	veiled chameleon serpentovirus B	VCSTV-B	*Chamaeleonidae*	MT997159
Tobaniviridae	*Serpentovirinae*	*Pregotovirus*	*Morelia tobanivirus 1*	Morelia viridis nidovirus	MVNV	*Pythonidae*	MF351889
Tobaniviridae	*Serpentovirinae*	*Infratovirus*	*Infratovirus latu*	serpentovirus-L25	SerTV-L25	*Colubridae*	MN161572
Tobaniviridae	*Serpentovirinae*	*Sertovirus*	*Sertovirus cona*	serpentovirus-C18	SerTV-C18	*Boidae*	MN161561
Tobaniviridae	*Serpentovirinae*	*Septovirus*	*Septovirus foka*	serpentovirus-K48	SerTV-K48	*Pythonidae*	MN161566
Tobaniviridae	*Serpentovirinae*	*Pregotovirus*	*Shingleback nidovirus 1*	shingleback nidovirus	ShNV	*Scincidae*	KX184715
Tobaniviridae	*Serpentovirinae*	*Pregotovirus*	*Pregotovirus heba*	Morelia viridis nidovirus	MoVNV	*Pythonidae*	MK182569
Tobaniviridae	*Serpentovirinae*	*Infratovirus*	*Hebius tobanivirus 1*	Hainan hebius popei torovirus	HpoToV	*Colubridae*	MG600028
Tobaniviridae	*Serpentovirinae*	*Lyctovirus*	*Lycodon tobanivirus 1*	Guangdong red-banded snake (Lycodon rufozonatus) torovirus		*Colubridae*	MG600030
Tobaniviridae	*Torovirinae*	*Torovirus*	*Torovirus banli*	Bangali torovirus	BanToV	*Camelidae*	MW561977
Tobaniviridae[a]	*Torovirinae*[a]	*unclassified*	*unclassified*	antelope torovirus	AToV	*Bovidae*	MZ438674

[a] Classification based on data presented in [36], which has not yet been ratified by International Committee for Taxonomy of Viruses.
[b] Classification based on data presented in [33], which has not yet been ratified by International Committee for Taxonomy of Viruses.
